# Ubiquitin-mediated control of seed size in plants

**DOI:** 10.3389/fpls.2014.00332

**Published:** 2014-07-11

**Authors:** Na Li, Yunhai Li

**Affiliations:** State Key Laboratory of Plant Cell and Chromosome Engineering, Institute of Genetics and Developmental Biology, Chinese Academy of SciencesBeijing, China

**Keywords:** seed size, seed development, ubiquitin, ubiquitin receptor, E3 ubiquitin ligase

## Abstract

Seed size in higher plants is an important agronomic trait, and is also crucial for evolutionary fitness. In flowering plants, the seed comprises three major anatomical components, the embryo, the endosperm and the seed coat, each with different genetic compositions. Therefore, seed size is coordinately determined by the growth of the embryo, endosperm and maternal tissue. Recent studies have revealed multiple pathways that influence seed size in plants. Several factors involved in ubiquitin-related activities have been recently known to determine seed size in *Arabidopsis* and rice. In this review, we summarize current knowledge of ubiquitin-mediated control of seed size and discuss the role of the ubiquitin pathway in seed size control.

## Introduction

In angiosperms, seed development is an important process in the life cycle. The seed contains the basic architecture of the plant and accumulates nutrients for germination and early seedling growth. The size of seeds is important for evolutionary fitness and stress responses. In addition, seed size is one of the most important components of seed yield. Crop plants have undergone selection for large seed size during domestication (Sundaresan, 2005; Song et al., 2007; Shomura et al., 2008; Fan et al., 2009).

Seed development begins with double fertilization in which one sperm cell fuses with the egg cell to form the diploid embryo, and the other sperm cell fuses with the central cell to give rise to the triploid endosperm (Chaudhury et al., 2001; Sundaresan, 2005). In monocots, the endosperm constitutes the major part of the mature seed. In most dicots, the endosperm grows rapidly in the beginning and is eventually consumed, and the embryo occupies most of the mature seed. Maternal integuments surrounding the developing embryo and endosperm develop into seed coat after fertilization (Chaudhury and Berger, 2001; Chaudhury et al., 2001). Therefore, the size of a seed is determined by the coordinated growth of the diploid embryo, the triploid endosperm and the maternal sporophytic integuments. However, it is only in recent decades that we have begun to identify some genes involved in seed size control (reviewed in Kesavan et al., 2013).

The ubiquitin pathway has been recently known to play an important part in plant seed size determination (Song et al., 2007; Li et al., 2008; Xia et al., 2013; Du et al., 2014). Ubiquitin is a conserved 76-amino-acid protein that is covalently attached to target proteins through the sequential action of three enzymes (Hershko and Ciechanover, 1998; Moon et al., 2004). Firstly, the ubiquitin activating enzyme (E1) forms a thioester bond with the C-terminal glycine of ubiquitin in an ATP-dependent manner and transfers the activated ubiquitin to a cysteinyl residue on the ubiquitin conjugating enzyme (E2). The E2 can either bind with the ubiquitin protein ligase (E3) to transfer ubiquitin directly to substrate proteins, or transfer ubiquitin to E3 in the case of HECT (homology to E6-AP C terminus) E3s, which then transfers it to the substrates (Pickart, 2001). In both cases, E3 defines the substrate specificity. Conjugation of a single ubiquitin to a substrate protein can modify its activity (Mukhopadhyay and Riezman, 2007); however, the ubiquitination process can repeat several times to attach new ubiquitin to the lysine residue of the conjugated ubiquitin on the substrate to form a polyubiquitin chain. The number and the location of ubiquitin molecules that are attached define the fates of the target. One of the famous forms of ubiquitylation with Lys-48 linked polyubiquitin chains often leads the substrate protein to the 26S proteasome for degradation (Vierstra, 2009).

The 26S proteasome is a multi-subunit protease that consists of a cylindrical 20S core particle (CP), capped on each end by a 19S regulatory particle (RP) (Finley, 2009). The 19S RP contains lid and base components, which recognize the ubiquitinated substrates, remove and recycle the ubiquitin moieties, unfold the target proteins and transport them into the central chamber of CP. The CP is a core protease in which proteolysis takes place and unfolded proteins are broken into peptides (Vierstra, 2009). During the degradation of polyubiquitinated proteins, ubiquitin chains linked to the substrates can be cleaved and recycled by deubiquitinating enzymes (DUBs) (Sadanandom et al., 2012). DUBs also generate free ubiquitin moieties from their initial translation products, or reverse the effects of ubiquitination by removing ubiquitin from the targets (Smalle and Vierstra, 2004). Thus, the ubiquitylation process in the cell is dynamic and highly controlled (Sadanandom et al., 2012).

Genomic analysis revealed that more than 1400 genes in *Arabidopsis thaliana* encode components of ubiquitin-26S proteasome pathway (Smalle and Vierstra, 2004). Ubiquitin-mediated signaling is involved in diverse aspects of plant life cycle, such as hormone signaling, circadian rhythm, pathogen responses, and abiotic stress responses (Sadanandom et al., 2012). Recently, several components of the ubiquitin pathway have been found to play critical roles in the regulation of seed and organ size (Table 1). In this review, we aim to summarize current knowledge on ubiquitin-mediated control of seed size and discuss the role of the ubiquitin pathway in seed growth.

**Table 1:** **List of ubiquitin-related proteins involved in seed size control**.

**Categories**	**Protein name**	**Species**	**Accession number**	**Reference(s)**
Ubiquitin receptors	DA1	*Arabidopsis*	AT1G19270	Li et al., 2008
	DAR1	*Arabidopsis*	AT4G36860	Li et al., 2008
E3 ligases	DA2	*Arabidopsis*	AT1G78420	Xia et al., 2013
	EOD1/BB	*Arabidopsis*	AT3G63530	Disch et al., 2006; Li et al., 2008
	DA2L	*Arabidopsis*	AT1G17145	Xia et al., 2013
	GW2	Rice	EF447275	Song et al., 2007
	TaGW2	Wheat	JN896622, JN896623	Su et al., 2011; Bednarek et al., 2012; Yang et al., 2012
	ZmGW2	Maize	EU968771, FJ573211, EU962093	Li et al., 2010
Other regulators	UBP15	*Arabidopsis*	AT1G17110	Liu et al., 2008; Du et al., 2014
	Rpt2a	*Arabidopsis*	AT4G29040	Kurepa et al., 2009
	SAMBA	*Arabidopsis*	AT1G32310	Eloy et al., 2012
	GW5	Rice	AB433345	Shomura et al., 2008; Weng et al., 2008

## Regulation of seed size by the ubiquitin receptors DA1 and DAR1

The *Arabidopsis da1-1* (DA means “large” in Chinese) mutant was isolated from a genetic screen for mutations that increase seed and organ size (Li et al., 2008). The *da1-1* mutant produced larger and heavier seeds than the wild type (Li et al., 2008). The increased seed size in *da1-1* was a result of enlargement of sporophytic integuments. In addition, *da1-1* plants formed large flowers, siliques, leaves and increased biomass compared with wild-type plants. *DA1* controls seed and organ growth by restricting cell proliferation. The *da1-1* mutation causes an arginine-to -lysine mutation in the position 358 of the DA1 protein (DA1^R358K^). In *Arabidopsis*, seven DA1-related (DAR) proteins share extensive amino acid similarity with DA1. DA1 homologs were also found in other plant species but not in animals, indicating a plant-specific mechanism to control seed and organ growth. Interestingly, the disruption of *DA1* or its closest family member *DAR1* with T-DNA insertions did not cause obvious seed and organ size phenotypes, while the simultaneous disruption of both *DA1* and *DAR1* resulted in large seeds and organs, indicating that *DA1* and *DAR1* act redundantly to restrict seed and organ growth. This genetic analysis also suggests that the mutant protein encoded by *da1-1* may have negative effects on DA1 and DAR1. Consistent with this notion, overexpression of a *da1-1* cDNA dramatically increased seed and organ size of wild-type plants.

*DA1* encodes a ubiquitin receptor containing two ubiquitin interacting motifs (UIMs) and a single zinc-binding LIM domain defined by its conservation with the canonical Lin-11, Isl-1, and Mec-3 domains (Li et al., 2008). UIM-containing proteins are characterized by coupled ubiquitin binding and ubiquitylation, which generally bring about monoubiquitylation of the ubiquitin receptor proteins. This, in turn, promotes the conformation change of the receptors, regulates their activity or binding capacity with other proteins, and initiates a signal cascade (Hicke et al., 2005). Considering that UIM domains of DA1 have the ubiquitin-binding activity, DA1 may be involved in ubiquitin-mediated signaling processes by coupled ubiquitin binding and ubiquitylation. On the other hand, ubiquitin receptors could bind polyubiquitinated proteins and mediate their degradation by the 26S proteasome (Verma et al., 2004). Thus, it is also possible that DA1 may interact with its polyubiquitinated substrates via UIM domains and facilitate their degradation.

## Regulation of seed size by the E3 ubiquitin ligases BB/EOD1, DA2, and GW2

There are two E1s, at least 37 E2s and more than 1300 E3s in *Arabidopsis* (Smalle and Vierstra, 2004). E3s function at the last step of the ubiquitylation cascade and recognize the specific substrates. E3s fall into two groups according to their conserved domains: HECT or RING (Really Interesting New Gene)/U-box type. The RING-type E3 ubiquitin ligases can act independently or as components of multi-subunit E3 complexes including SCF (SKP1-CULLIN-F-box), CUL3 (CULLIN 3)- BTB/POZ (Bric a brac, Tramtrack and Broad complex/Pox virus and Zinc finger), CUL4-DDB1 (UV-Damaged DNA Binding Protein 1) and APC (Anaphase Promoting Complex) (Mazzucotelli et al., 2006). Currently, several RING-type E3 ubiquitin ligases have been identified as key factors of seed size control in dicot and monocot plants.

**Figure 1:**
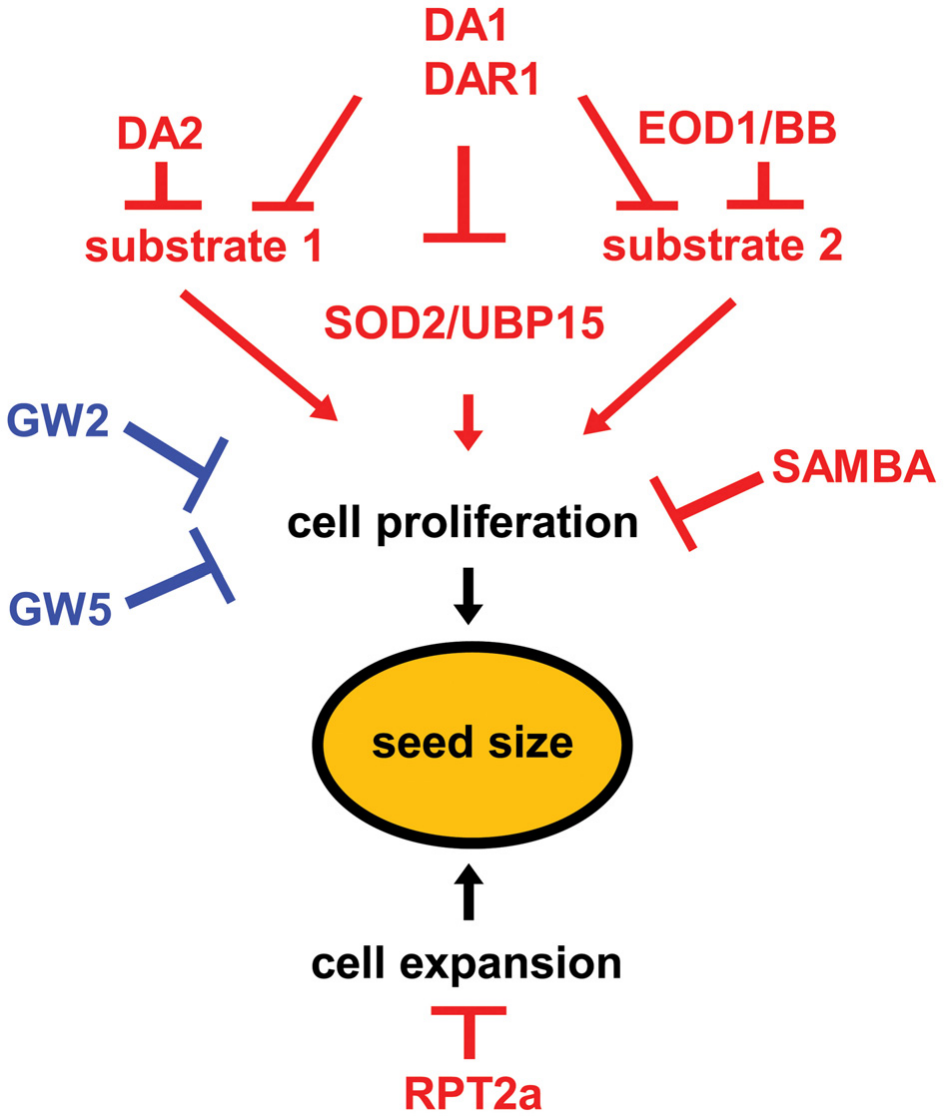
**A genetic and molecular framework for ubiquitin-mediated control of seed size**. DA1 and DAR1 act redundantly to restrict cell proliferation. DA1 and DA2 may control cell proliferation by suppressing a common substrate (substrate 1). Similarly, DA1 and EOD1 may regulate cell division through a common target (substrate 2). DA1 acts upstream of UBP15 and modulates its stability to control cell proliferation. GW2, GW5 and SAMBA control seed size by restricting cell proliferation, respectively. RPT2a regulates seed size by limiting cell expansion. The seed size regulators in *Arabidopsis* and rice are shown as red and blue, respectively.

Two RING-type E3 ubiquitin ligases, DA2 and Big Brother (BB)/Enhancer of DA1 (EOD1), were identified as negative regulators of seed size in *Arabidopsis* (Li et al., 2008; Xia et al., 2013). Loss-of-function *da2-1* and *eod1/bb* mutants shared similar phenotypes, such as large organs and increased biomass. Overexpression of either *DA2* or *BB/EOD1* resulted in a reduction in organ size (Disch et al., 2006; Xia et al., 2013). In addition, both EOD1 and DA2 act maternally to regulate seed size by restricting cell proliferation in the integuments of ovules and developing seeds (Li et al., 2008; Xia et al., 2013), suggesting that these two E3 ubiquitin ligases may share similar mechanisms in seed size control. Importantly, both the *eod1* and *da2-1* mutations synergistically enhance the seed size and weight phenotypes of *da1-1*, suggesting that both EOD1 and DA2 may function with DA1 to control seed size by modulating the activity of common downstream targets. However, genetic analyses show that *DA2* and *EOD1* function independently to control seed size (Figure 1) (Xia et al., 2013), suggesting that DA2 and EOD1 may target distinct growth stimulators for degradation, with common regulation via DA1. The synergistic effects could result from the simultaneous disruption of two components of a protein complex (Perez-Perez et al., 2009; Lanctot et al., 2013). It has been demonstrated that the ubiquitin receptor DA1 interacts with the E3 ligase DA2 through its C-terminal region (Xia et al., 2013), and the UIM domains of DA1 can bind ubiquitin (Li et al., 2008). Thus, it is likely that the interaction between DA1 and DA2 helps DA1 to bind the ubiquitinated substrates of DA2 and facilitate their degradation by the proteasome.

In rice (*Oryza sativa*), a quantitative trait locus (QTL) for *GRAIN WIDTH AND WEIGHT2* (*GW2*) encodes a RING-type E3 ubiquitin ligase (Song et al., 2007). Loss-of-function *GW2* allele caused wide spikelet hulls and accelerated grain milk-filling rates, resulting in increased grain width, weight and yield. The naturally occurring WY3 allele of *GW2* encoding a truncated version of the protein with a 310-amino acid deletion produced wide and heavy grains due to increased cell proliferation in spikelet hulls. In contrast, transgenic rice plants overexpressing *GW2* formed smaller and lighter grains than wild-type plants. Thus, GW2 might negatively affect the level or the activity of factors promoting cell proliferation. Interestingly, GW2 shares significant similarity with *Arabidopsis* DA2 and DA2-like protein (DA2L) (Xia et al., 2013). Overexpression of *GW2* in *Arabidopsis* resulted in small seeds and organs, as it has been observed in *35S:DA2* and *35S:DAL2* transgenic plants (Xia et al., 2013), indicating a possible conserved function in *Arabidopsis* and rice. The RING domain of GW2 is characterized by a Cys at metal ligand position 5 and a His at metal ligand position 6 (C5HC2) (Song et al., 2007). This feature is shared by the RING domain of maize, wheat, yeast and fungal homologs. Although the spacing of the Cys residues in the RING domain of DA2 is similar to that in the RING domain of GW2, the RING domain of DA2 or its dicot homologs lacks a conserved His residue that is replaced by Asn (Asn-91) (Xia et al., 2013). Biochemical and genetic analyses showed that this amino acid (Asn-91) is not required for DA2 E3 ligase activity and the roles of DA2 in seed size control, suggesting that the RING domain of DA2 might be a variant of that found in GW2.

In wheat (*Triticum aestivum*), there are three *GW2* homologs (originating from A, B, and D genomes, respectively) (Su et al., 2011). Analysis of modern varieties showed that *TaGW2-6A* Hap-6A-A is a superior allele for grain size. Varieties with *TaGW2-6A* Hap-6A-A allele had higher mean grain width than those with Hap-6A-G. This effect was due to the higher expression level of *TaGW2* in the varieties with Hap-6A-G allele, indicating that the expression level of *TaGW2* was negatively correlated with grain width. Meanwhile, a single base (T) insertion in the eighth exon of *TaGW2-6A* was detected in a large-kernel wheat variety, Lankaodali. This mutation produced a truncated protein, indicating that TaGW2-6A has a negative effect on grain size. In contrast, another report showed that overall down-regulation of *TaGW2* by RNA interference resulted in decreased grain size and weight, suggesting that *TaGW2* may positively regulate grain size (Bednarek et al., 2012). Further studies are needed to elucidate the role of TaGW2 in grain size control.

*ZmGW2-CHR4* and *ZmGW2-CHR5*, two homologs of the rice *GW2*, have been found in maize (*Zea mays*) (Li et al., 2010). These two loci were located on duplicated maize chromosomal regions that have co-orthologous relationships with the rice region containing *GW2*. Single nucleotide polymorphism (SNP) in the promoter region of *ZmGW2-CHR4* was significantly associated with kernel width and one-hundred kernel weight, and the expression level of *ZmGW2-CHR4* was negatively correlated with kernel width. Similarly, *ZmGW2- CHR5* also affected kernel width (Li et al., 2010).

## Regulation of seed size by the ubiquitin-specific protease UBP15

*SUPPRESSOR2 OF DA1* (*SOD2*) encodes UBIQUITIN-SPECIFIC PROTEASE15 (UBP15), which is a deubiquitinating enzyme (Liu et al., 2008; Du et al., 2014). UBP15 contains a ubiquitin-specific protease (UBP) domain that is required for deubiquitination activity, and a signature MYND-type zinc finger domain (Zf-MYND) that is supposed to function in protein-protein interaction. *sod2/ubp15* mutants were identified as suppressors of *da1-1*. *sod2/ubp15* plants produced small leaves, flowers and seeds, whereas plants overexpressing *UBP15* formed large seeds and organs, indicating that UBP15 is a positive regulator of seed and organ growth. UBP15 functions to regulate seed size by promoting cell proliferation in maternal integuments of ovules and developing seeds. Genetic analyses show that *ubp15* is epistatic to *da1-1* with respect to seed size, suggesting that UBP15 acts downstream of DA1 to promote seed growth (Figure 1). UBP15 protein is stabilized by adding proteasome inhibitor MG132, suggesting that UBP15 is degraded by the 26S proteasome. Furthermore, DA1 physically interacts with UBP15 and modulates its stability. It is likely that the ubiquitin receptor DA1 targets UBP15 and mediates its degradation by the proteasome. However, UBP15 acts independently of the E3 ubiquitin ligases BB/EOD1 and DA2 to control seed size (Figure 1), indicating that UBP15 is not the substrate of the E3 ubiquitin ligases DA2 or EOD1, and also suggesting that other E3 ligase(s) might be involved in proteasome-dependent degradation of UBP15.

The *Arabidopsis* genome encodes 27 UBPs, which were clustered into 14 subfamilies (Yan et al., 2000). The *UBP15* subfamily contains five genes (*UBP15-19*). Although loss of function in the *UBP16* gene had no obvious growth defects, the *ubp16* mutation enhanced the organ growth phenotypes of *ubp15*, indicating that UBP15 and UBP16 function redundantly to control organ size (Liu et al., 2008). It would be interesting to investigate whether UBP16 is involved in seed size control. It is also a worthwhile challenge to know if DA1 could interact genetically and physically with UBP16 and target it for degradation.

## Regulation of seed size by RPT2a, a subunit of the 26S proteasome

The RP of the 26S proteasome is composed of a lid containing non-ATPase subunits (RPN3, 5–9, and 11–12) and a base consisting of six related AAA-ATPases (RPT1-6) and three non-ATPase subunits (RPN1, 2, and 10) (Smalle and Vierstra, 2004). One of the base subunits, the regulatory particle RPT2, has been found to affect seed size (Kurepa et al., 2009). There are two RPT2 homologs (RPT2a and RPT2b) in *Arabidopsis*, which share 98.8% identity in amino acid sequences. Loss-of-function of RPT2a caused a weak defect in 26S proteasome activity and led to enlargement of most organs including seeds. The size of cells in *rpt2a* mutants was increased compared with that in the wild type, while the number of cells in *rpt2a* mutants was reduced, suggesting a possible compensation mechanism between cell proliferation and cell expansion. It is plausible that the RP of 26S is required for the degradation of the positive regulators of cell expansion, thereby influencing seed size.

## Regulation of seed size by SAMBA, a plant- specific APC/C regulator

Plant organ growth is coordinated by cell division and cell expansion. Cell cycle progression is controlled by the degradation of essential cell cycle regulators such as securin or cyclins (De Veylder et al., 2003). In plants, A- and B-type cyclins are specifically recognized by a multi-subunit E3 ubiquitin ligase complex called anaphase-promoting complex/cyclosome (APC/C) (Heyman and De Veylder, 2012). The cyclins are then subjected to proteolysis by the 26S proteasome, and this promotes the mitotic progression. The activities of plant APC/C are regulated by different activating proteins or inhibitors including CELL DIVISION CYCLE 20 (CDC20), CDC20 HOMOLOGY1/CELL CYCLE SWITCH 52 (CDH1/CCS52), ULTRAVIOLENT-B-INSINSITIVE4 (UVI4), UVI4-like/OMISSION OF SECOND DIVISION1/GIGAS CELL1 (UVI4/OSD1/GIG1), and SAMBA. SAMBA is a plant-specific APC/C regulator that plays a role in seed size control (Eloy et al., 2012). In *Arabidopsis thaliana*, *SAMBA* is expressed in developing seeds and during early plant development stages. Loss of function of *SAMBA* stabilizes the A-type cyclin CYCA2;3 and promotes cell proliferation and endoreduplication, resulting in large seeds and organs. The yeast two-hybrid assay showed that SAMBA specifically interacts with A-type cyclins. These results indicate that SAMBA targets A-type cyclins for APC/C- mediated degradation and acts as a negative regulator of seed growth.

## Regulation of seed size by GW5

Rice *GW5* is a major QTL that controls rice grain width and weight (Shomura et al., 2008; Wan et al., 2008; Weng et al., 2008). Fine mapping of this locus uncovered that a 1212-bp deletion including the *GW5* gene is correlated with increased grain width (Shomura et al., 2008; Weng et al., 2008). Genotyping analysis of rice cultivars revealed that an intact *GW5* was detected in the slender-grain rice, whereas the 1212-bp deletion was observed in the wide-grain lines, suggesting a strong artificial selection during breeding (Shomura et al., 2008). *GW5* encodes a nucleus-localized protein of 144 amino acids with a predicted nuclear localization signal and an arginine-rich domain. GW5 interacted with polyubiquitin in a yeast two-hybrid assay (Weng et al., 2008), suggesting that GW5 might be involved in the ubiquitin-proteasome pathway to regulate cell division during seed development. As the E3 ubiquitin ligase GW2 also controls glume cell division and grain width, it has been hypothesized that GW5 and GW2 might act in the same pathway. However, genetic analyses showed that plants pyramiding *gw2* and *gw5* exhibited an enhanced phenotype of grain width compared with those carrying one of the two major QTLs (Ying et al., 2012), suggesting that they may act in different pathways or function in a same complex to regulate rice grain size.

## Challenges and future perspectives

During the past decade, several factors involved in ubiquitin-related activities have been identified to influence seed size in plants, indicating that the ubiquitin pathway plays an important role in seed size control. Interestingly, most of these factors affect not only seed size but also organ growth. For example, *da1* mutant showed large seeds, leaves, and flowers (Li et al., 2008), whereas *sod2* mutants produced small seeds and organs (Du et al., 2014), suggesting a possible link between seed size control and organ growth. By contrast, several other mutants with large organs formed normal-sized seeds (Horiguchi et al., 2005; White, 2006; Xu and Li, 2011), implying that seed and organ size is not always positively related. These results suggest that seeds and organs may possess both common and distinct pathways to regulate their respective size.

Our current knowledge of ubiquitin-mediated control of seed size is rather fragmented, relying on several seemingly independent pathways full of gaps (Figure 1). One of the major challenges in the future is to define the molecular function of the known factors in seed size control. For example, what are the specific targets of the ubiquitin receptors and the E3 ubiquitin ligases? How are the activities of these receptors and E3 ligases regulated? Thus, identification of their interacting proteins and downstream targets by biochemical and genetic approaches will help fill up the major gaps in each pathway and understand the molecular mechanisms of these factors in seed size control. To identify novel ubiquitin-related factors in seed size control, both forward and reverse genetic approaches could be used. Genetic screens for modifiers of the known genes will help identify downstream targets of the ubiquitin receptors or the E3 ligases. The use of the newly developed genome editing technology (reviewed in Gaj et al., 2013) will greatly facilitate the functional characterization of candidate ubiquitin-related genes involved in seed size regulation. On the other hand, systems biology approaches, such as transcriptomic, proteomic, and metabolomics analysis, should yield novel insights into the molecular networks of ubiquitin-mediated control of seed size.

### Conflict of interest statement

The authors declare that the research was conducted in the absence of any commercial or financial relationships that could be construed as a potential conflict of interest.
